# The Intestinal Redox System and Its Significance in Chemotherapy-Induced Intestinal Mucositis

**DOI:** 10.1155/2022/7255497

**Published:** 2022-05-09

**Authors:** Qing-Qing Yu, Heng Zhang, Yujin Guo, Baoqin Han, Pei Jiang

**Affiliations:** ^1^Laboratory of Biochemistry and Biomedical Materials, College of Marine Life Sciences, Ocean University of China, Qingdao 266003, China; ^2^Jining First People's Hospital, Jining Medical College, Jining 272000, China; ^3^Department of Laboratory, Shandong Daizhuang Hospital, Jining 272051, China; ^4^Laboratory for Marine Drugs and Bioproducts of Qingdao National Laboratory for Marine Science and Technology, Qingdao 266235, China

## Abstract

Chemotherapy-induced intestinal mucositis (CIM) is a significant dose-limiting adverse reaction brought on by the cancer treatment. Multiple studies reported that reactive oxygen species (ROS) is rapidly produced during the initial stages of chemotherapy, when the drugs elicit direct damage to intestinal mucosal cells, which, in turn, results in necrosis, mitochondrial dysfunction, and ROS production. However, the mechanism behind the intestinal redox system-based induction of intestinal mucosal injury and necrosis of CIM is still undetermined. In this article, we summarized relevant information regarding the intestinal redox system, including the composition and regulation of redox enzymes, ROS generation, and its regulation in the intestine. We innovatively proposed the intestinal redox “Tai Chi” theory and revealed its significance in the pathogenesis of CIM. We also conducted an extensive review of the English language-based literatures involving oxidative stress (OS) and its involvement in the pathological mechanisms of CIM. From the date of inception till July 31, 2021, 51 related articles were selected. Based on our analysis of these articles, only five chemotherapeutic drugs, namely, MTX, 5-FU, cisplatin, CPT-11, and oxaliplatin were shown to trigger the ROS-based pathological mechanisms of CIM. We also discussed the redox system-mediated modulation of CIM pathogenesis via elaboration of the relationship between chemotherapeutic drugs and the redox system. It is our belief that this overview of the intestinal redox system and its role in CIM pathogenesis will greatly enhance research direction and improve CIM management in the future.

## 1. Introduction

Chemotherapy-induced intestinal mucositis (CIM) is defined as an intestinal disorder caused by chemotherapy in ESMO (European Society for Medical Oncology) [1, 2]. Based on the differences in chemotherapeutic drugs, the clinical incidence of CIM can be up to 40-76% [3]. However, the recommended drugs in ESMO, such as ranitidine, octreotide, and omeprazole, exhibit poor efficacy [2], which suggests the need for clarification of the underlying mechanism behind CIM pathogenesis.

The following describes a typical CIM presentation in a five-phase model: (1) initiation, (2) upregulation and message generation, (3) signaling and amplification, (4) ulceration, and (5) healing [4–6]. Existing studies revealed that ROS is rapidly produced in the initial stage, at the time when chemotherapeutic drugs cause direct damage to the intestinal mucosa cells and produce mitochondrial dysfunction. Although the intestinal mucosa is normal at this stage, the cascade reaction that eventually results in submucosal damage has already initiated [7]. However, the mechanism involving intestinal oxidation-reduction (redox) system, ROS production, and induction of intestinal mucosal injury within CIM is not fully understood.

This review has two segments, the first segment details intestinal physiological redox pathways, and the second summarizes available information on CIM. To reiterate, our first segment discusses the intestinal redox system, including the composition and regulation of redox enzymes, as well as reactive oxygen species (ROS) generation and its intestinal regulation. This section details the redox system-mediated modulation of the physiological intestinal function. The second segment discusses the redox system-mediated modulation of CIM pathogenesis via elaboration of the relationship between chemotherapeutic drugs and the redox system and the involvement of ROS in CIM development. We also discussed the significance of intestinal redox system in CIM.

## 2. Overview of Oxidation-Reduction (Redox) System in the Intestine

The digestive system is responsible for digesting and absorbing nutrients and possesses unique gut morphology. The interaction between intestinal flora and intestinal redox system is not discussed in this review. Since the intestine links an organism with its outside environment, it automatically facilitates protection against luminal toxic agents, which is also primarily done by the redox system. Thus, it is necessary to elucidate the composition and regulation of the intestinal redox system to explain the physical mechanism of the digestive system, particularly in terms of absorption and defense function.

## 3. Enzyme Related to OS Generation in the Intestine

### 3.1. Nicotinamide Adenine Dinucleotide Phosphate (NADPH) Oxidase (NOX)

**Figure 1:**
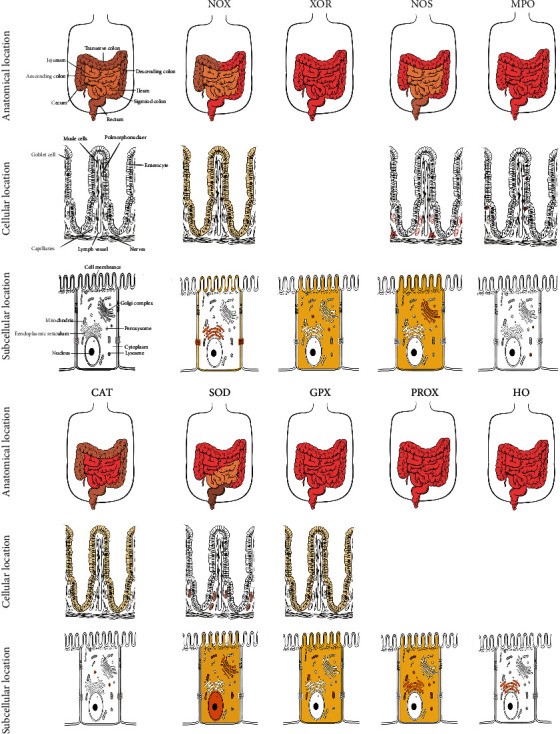
The anatomical, cellular, and subcellular locations of the intestinal oxidoreductases. The anatomical location of oxidoreductase is shown in red color (the top line). The cellular and subcellular locations of oxidoreductases are represented by yellow or brown color.

NOX belongs to the membranal flavoprotein NADPH-dependent oxidoreductase family, and it accelerates oxygen (O_2_) reduction to form superoxide (O^2•−^) (Table 1). Thus far, six cytochrome homologs have been identified within the subunit of the phagocyte NOX, namely, NOX1, NOX3, NOX4, NOX5, DUOX1, and DUOX2. Among them, NOX1 is ubiquitously present in the colon (Figure 1) [8–10]. However, the expression is scarce in the proximal colon and relatively high in the distal colon [11, 12]. Using in situ hybridization, it was shown that NOX1, within the colon wall, was evenly distributed between the apical surface and crypts [12] or exhibited concentrated expression in the lower regions of the crypts [13]. Moreover, the largest expression is evident on the mucosal surface. DUOX2 is also present in the distal gastrointestinal (GI) tract; specifically, it can be found in the cecum, sigmoidal colon, and rectal glands [14–16].

In terms of structure, NOX has six N-terminal transmembranal *α*-helices, a flavin adenine dinucleotide- (FAD-) docking pocket, and a C-terminal NADPH-docking pocket. Multiple reports indicated that the NOX1 molecular mass ranges between 55 and 60 kDa [17–19]. The human NOX1 gene 5′-region is known to possess elements that specialize in interaction with signal transducers and activators of transcription (STATs), interferon regulatory factor (IRF), AP-1, NF-*κ*B, CREB, CBP/p300 elements [20], and GATA factors [21]. NOX1 overexpression in intestinal epithelial cells is strongly associated with GATA-interacting sites [21], whereas interferon-*γ* overexpression is modulated by the interaction between stimulated STAT1 dimers and *γ*-activated sequence (GAS) element [20]. DUOX enzymes typically undergo glycosylation. DUOX1 and DUOX2 exist in one of two N-glycosylation states. One is an elevated mannose glycosylation, which is typically observed in the endoplasmic reticulum (ER), and presents as an 180 kDa protein band on gel electrophoresis. The second is a complete glycosylation status, which is typically identified on the plasma membrane, and is represented by a 190 kDa protein band on gel electrophoresis [22, 23]. Based on carbohydrate content analysis of membranal DUOX, the presence of particular oligosaccharides involved in Golgi apparatus (GA) processing was identified [24]. DUOX2 is typically located all over the GI tract, namely, the duodenum, colon, and cecum [14, 16], and its transcription is triggered by interferon-*γ* in response to the spontaneous differentiation of postconfluent Caco-2 cells [14].

**Figure 2:**
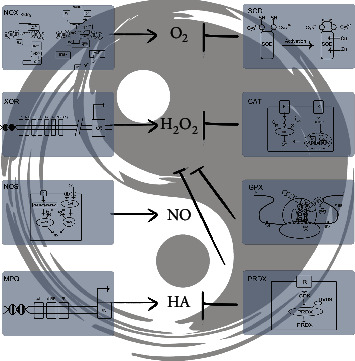
Intestinal redox regulation. OS regulation via oxidoreductases maintains normal physiological intestinal homeostasis, similar to the balance of the “Tai Chi” theory. The regulation of NOX, CAT, PRDX, and NOS was mainly at the posttranslational level; the others were at the translational level.

NOX is activated after the assembly of additional proteins, such as membranal p22^phox^, which stabilizes NOX proteins and interacts with cytosolic agents and proteins p47^phox^, p67^phox^, small GTPase Rac, and p40^phox^ (Figure 2). Synergistically, these proteins activate NOX. Once stimulated, p47^phox^, along with the bound p47^phox^, relocates to the membrane. p47^phox^, in this scenario, is thought to behave as an organizer. Upon translocation, p67^phox^ directly binds and activates NOX2. Thus, p67^phox^ behaves as an activator [25]. Simultaneous to this process, GTP-binding protein Rac also translocates to the cell membrane, whereby it activates NOX2 [26]. Lastly, the newly discovered subunit p40^phox^ [27] is thought to play a nonessential, but regulatory role. The newly discovered subunits were termed as NOXO1 (NOX organizer = p47^phox^ homolog) and NOXA1 (NOX activator = p67^phox^ homolog). Interestingly, even though the expression systems employing mouse proteins demonstrated potent constant activity of the NOX1/NOXO1/NOXA1 network, using human proteins resulted in only diminutive activity. NOX1 activation, in such cases, was only possible via introduction of PKC activator phorbol 12-myristate 13-acetate (PMA) [25, 28]. Emerging evidences also suggest that small GTPase Rac modulates NOX1 activity [25, 29–32]. Rac interacts with the TPR domain of the activator NOXA1 [25, 29, 30], but, similar to NOX2, the Rac-mediated NOX1 stimulation may require two steps and physical interaction with NOX1.

### 3.2. Xanthine Oxidoreductase (XOR)

XOR commonly represents two interconvertible states of the same enzyme: dehydrogenase (XDH) and oxidase (XO) [33]. XOR oxidizes hypoxanthine and xanthine to form xanthine and uric acid, respectively, as part of the purine degradation process. In humans, XOR is primarily located in the liver and intestine [34], whereas other human organs display minute XOR activity (Figure 1) [35, 36]. Within cells, XOR is primarily found in the cytoplasm and sometimes in organelle membranes like peroxisomes (Table 2)

Upon transcription and translation, XOR forms XDH, a ~300 kDa homodimer that consists of four redox regions per subunit: one molybdenum cofactor (Mo-co), one flavin adenine dinucleotide (FAD) location, and two Fe_2_S_2_ locations (Table 1) [37]. It is a critical enzyme in the last step of endogenous and exogenous purine metabolism. XOR typically exists in two interconvertible forms: XO promotes O_2_ reduction in one electron transfer. In contrast, although XDH reduces O_2_, it prefers nicotinamide adenine dinucleotide (NAD^+^) reduction via two electron transfers [35, 38]. Both forms also oxidize hypoxanthine and xanthine to UA via binding with Mo at the docking site, where it donates two electrons, thereby reducing it from Mo^6+^ to Mo^4+^. Mo^6+^ is then formed again via transfer of two electrons from Mo^4+^ to flavin adenine dinucleotide (FAD) using an iron sulfur center. This generates flavin adenine dinucleotide hydroquinone (FADH2), which, in turn, donates electrons to either O_2_ forming O^2•−^ anions and hydrogen peroxide (H_2_O_2_) or to NAD^+^, thus generating nicotinamide adenine dinucleotide hydride (NADH). Given these evidences, the XO-mediated catabolic reactions occur in conjunction with ROS generation [39].

Despite extensive studies on XOR biochemistry, little is reported on the different types of XOR modulation (Figure 2). The human XDH gene resides on the p22 band of chromosome 2, and its protein comprises of multiple potential docking sites for translational modification, namely, four CCAAT/enhancer docking sites, three IL-6 responsive elements (RE), and a NF-*κ*B site, as well as TNF-*α*, interferon-*γ*, and interleukin-1 REs [38, 40]. In addition, although multiple studies reported severe hypoxia-mediated modulation of both transcriptional and posttranslational XOR [41–43], moderate hypoxia (10% O_2_) also induces marked upregulation of XOR levels, activity, release from endothelial cells, and XO-based ROS generation [44].

### 3.3. Nitric Oxide Synthase (NOS)

NOS is a collection of enzymes that promote nitric oxide (NO) synthesis from the nitrogen residue of L-arginine, under regulation of NADPH and molecular O_2_. The NOS directly interacts with FAD, flavin mononucleotide (FMN), heme, tetrahydrobiopterin (BH4), and calmodulin (Table 1). Till now, three NOS versions were identified in mammals. Among them, nNOS (i.e., type I, NOS-I, and NOS1) was the first discovered isoform, and it predominately resides within neurons. The next isoform is iNOS (i.e., type II, NOS-II, and NOS2), and it can be induced in many cells and tissues. The last isoform is eNOS (i.e., type III, NOS-III, and NOS3), and it was initially recorded in vascular endothelial cells. In addition to this, nNOS and eNOS were also identified in the intestinal myenteric neurons and enteric arterioles [45, 46]. It was reported that the iNOS isoform is activated by inflammatory cytokines employing the NF-*κ*B pathway. Moreover, in 2012, while examining NF-*κ*B signaling in colitis, Gochman et al. reported relatively high iNOS expression in human colitis tissue [47] (Figure 1).

Structurally, the three reported isoforms are comparable to one another. All are made of dimmers of two similar subunits [48, 49]. In addition, each monomer consists of three domains, namely, reductase, oxygenase, and calmodulin-binding domains. The reductase domain harbors docking sites for FMN, FAD, and NADPH. In contrast, the oxygenase portion interacts with tetrahydrobiopterin (BH4). The primary function of the reductase portion, which comprises the functional groups FMN and FAD, is to donate electrons from NADPH to the oxygenase of the corresponding subunit. Subsequently, the calmodulin-binding domain regulates all NOS isoform activities [50]. All NOS isoforms promote the same reaction. At the initial step, NOS catalyzes L-arginine oxidation to form an intermediate molecule, N-hydroxy-L-arginine. This is next oxidized to L-citrulline to generate NO [51].

NO, an end-result of an NOS-based reaction, modulates both NOS expression and activity (Figure 2). Its modulation of amino acid residues to form an S-nitroso group reversibly suppresses NOS activity [52]. Scientists demonstrated a negative NO feedback loop using S-nitrosylation [53]. In this process, both NOS1 and NOS2 undergo S-nitrosylation. However, the dynamic modulation of their physiological function using this process needs further investigation. Moreover, phosphorylation of nNOS and eNOS isoforms modulates NOS action. Fluid shear stress phosphorylates eNOS, thus enhancing noncalcium-related NOS action [54, 55]. Multiple reports revealed that protein kinase Akt-mediated phosphorylation of the Ser^1179^ residue of eNOS [56, 57] augments electron flux via the reductase domain, thereby increasing NO production [58]. Alternately, CaM-dependent kinase-mediated Ser^847^ phosphorylation of nNOS drastically reduces NOS activity [59, 60].

### 3.4. Myeloperoxidase (MPO)

MPO is a component of polymorphonuclear leukocytes that protects hosts from foreign pathogens. In fact, it possesses proven microbicidal activity against a myriad of organisms. In activated PMN, MPO promotes hypohalous acid production. Under physiologic conditions, it triggers hypochlorous acid synthesis, as well as other toxic intermediates that greatly augment PMN microbicidal activity. Under regulation of H_2_O_2_ and a halide, such as chloride, bromide, or thiocyanate, MPO triggers reactive O_2_ intermediate production, which includes hypochlorous (HOCl), hypobromous, and hypothiocyanous acids, respectively (Table 1) [61, 62]. In activated neutrophils in the peripheral blood and tissues (Figure 1), MPO is secreted into phagolysosomes and the extracellular space (Table 2). Moreover, MPO mediates regional tissue damage and triggers inflammation in a myriad of intestinal inflammatory conditions [63–66].

Recent reports examined the biosynthesis and structure of MPO [67–69]. In brief, MPO originates from a singular gene in chromosome 17. Once translated, it forms an 80 kDa protein, which undergoes cleavage to generate a signal peptide. The N-linked glycosylation of the signal peptide, along with subsequent deglucosylation produces a 90 kDa enzymatically inactive apoproMPO. However, with heme introduction, apoproMPO is altered to the enzymatically active proMPO, which briefly interacts with calnexin. The proteolytic cleavage of proMPO removes the N-terminal 125 amino acid proregion, and a 72-75 kDa protein remains. A second proteolytic cleavage produces the heavy 59 kDa *α*-subunit and light 13.5 kDa *β*-subunit of MPO. This, in turn, forms a heavy-light protomer. A mature MPO possesses a molecular mass of 150 kDa and has a pair of heavy-light protomers, whereby the heavy subunits are connected via a disulfide bond.

The MPO gene sits on chromosome 17q22 and possesses twelve exons [70]. MPO is primarily expressed during the advanced myeloblast to the promyelocyte phases of normal myeloid formation [71, 72], and MPO expression is silenced once cells begin to differentiate [73]. The MPO gene expression is promoted by the excessive demethylation of the 5′ flanking region, which essentially opens up the chromatin structure for transcription to ensue [74, 75]. Upon transcription into mRNA, alternative splicing cleaves the original mRNA to produce secondary mRNA measuring 3.6 and 2.9 kB [76]. The MPO gene expression is also modulated by the transcription factor (TF) AML1, and, therefore, AML1 site integrity is crucial to the transcription of the gene (Figure 2) [77, 78]. The Reynolds' study recognized an allelic polymorphism, -463G/A, in the promoter region of the MPO gene [79]. It harbors an Alu receptor response element (AluRRE), which can interact with multiple nuclear receptors, including Sp1[79, 80]. In presence of an intact -463G Sp1 site, the MPO transcription rate increases by 25-fold, compared to the -463A Sp1 location.

## 4. Antioxidative Generation-Related Enzyme in the Intestine

### 4.1. Catalase (CAT)

The oxidoreductase CAT accelerates the splitting of H_2_O_2_ to water and O_2_. There are three categories of CATs: normal CATs or monofunctional (for example, mammal type CATs), bifunctional CAT (such as, peroxidases), and pseudo CAT. The human CAT has a standard monofunctional heme-harboring CAT, with a prosthetic ferric protoporphyrin IX group that interacts with H_2_O_2_. It is primarily found in peroxisomes, with a molecular mass of about 220-240 kDa [81]. In mammalian tissues, liver and erythrocytes exhibit the largest CAT activity, kidney and adipose tissue show relatively elevated activity, lung and pancreas display intermediate activity, and heart and brain show very little activity (Figure 1). Moreover, CAT forms particles within the small intestinal epithelium [82].

The human CAT is a tetrameric protein, and each of its subunits is further categorized into four domains, namely, the N-terminal threading arm, C-terminal helices, wrapping loop, and *β* barrel [81, 83]. Each subunit contains a hydrophobic core that has eight stranded *β* barrels encircled by *α*-helices. Each subunit polypeptide chain contains residues 4–502. In addition, subunit B also has residue Glu503. The N-terminal threading arm (residues 5–70) forms a bridge between two subunits by forming a long encircled loop (residues 380–438) surrounding the other subunit. Lastly, a helical domain associated with the *β* barrel possesses four c-terminal helices (*α*16, *α*17, *α*18, and *α*19) and four helices made from residues between *β*4 and *β*5 (*α*4, *α*5, *α*6, and *α*7).

CAT is typically modulated via transcription and posttranscriptional factors (Figure 2). The mammalian CAT promoter is heavily conserved. Therefore, it enables an effective interaction with TFs NF-Y, Sp1, and WT1/Egr in the core domain. It is also reported that the Fox family members, modulated by the Akt/PKB axis, also contain highly conserved docking sites in vertebrate CAT promoters [84]. Posttranslational modifications like phosphorylation (Ser^167^) [85], glycation [86], and acetylation [87] reduce CAT activity. Alternately, CAT covalently interacts with p53 [88], and ATM (ataxia telangiectasia mutated) [89] proteins promote CAT enzymatic activity. Furthermore, CAT modulation also includes structural alterations. H_2_O_2_, along with many other chemicals, abrogates CAT activity [90–92]. The active CAT domain is not directly modulated by H_2_O_2_ oxidation. Instead, it induces conformational alterations (a catalysis requirement) via amino acid residue oxidation. Exogenous nitric oxide is also known to inhibit CAT activity; however, in this case, the effect is reversible [93].

### 4.2. Superoxide Dismutase (SOD)

SOD triggers the conversion of O^2•−^ and hydrogen into molecular O_2_ and H_2_O_2_, thereby protecting the cell from toxic O^2•−^ concentrations. There are three mammalian SOD isoforms, namely, SOD1, SOD2, and SOD3 [94]. SOD1 and SOD2 are ubiquitously found in all cells. Elevated SOD3 levels were detected in select tissues, including blood vessels, lung, kidney, and heart [95]. The Cu/Zn SOD SOD1 is primarily cytoplasmic. However, it can be found in the nucleus, lysosome, peroxisome, and mitochondrial intermembranal space as well [96]. The MnSOD2 resides in the mitochondrial matrix [97]. Lastly, the Cu/Zn SOD SOD3 is typically released into the extracellular space (Table 2) [98].

MnSOD is present both as a tetramer and dimer, and all information related to its structures (reduced, oxidized, and specifically mutated enzymes) is available in the Protein Database. Eukaryotic MnSODs (for example, humans) are typically tetrameric. The monomeric structure has two domains, an N-terminal (primarily) *α*-helical region and a C-terminal region with a small *β* sheet and *α*-helices[99–101]. The difference between the two types of MnSODs is that, in tetrameric MnSODs, the N-terminal region consists of long *α*-helices that form a hairpin structure [102]. MnSODs possess varied cellular activities at both elevated and reduced O^2•−^ concentrations. This is known as “gating,” and is typically assessed via a range of stopped-flow and pulse radiolysis investigations [103, 104]. Interestingly, at high concentrations, O^2•−^ undergoes a biphasic process, whereby there is a rapid loss of O^2•−^ (burst phase), followed by a reduced loss rate (zero-order phase). Moreover, during proton delivery, there is a reversible isomerization, whereby the bound peroxy moiety is isomerized [104]. It was suggested that residues, such as Tyr^34^ [105], His^30^ [106], Glu ^143^ [107], and Phe^66^ [108], modulate this process.

The eukaryotic SOD1 is a 32 kDa homodimer, whereby all subunits carry one copper- and one zinc-interacting region close to one another, and it has a disulfide bond between Cys^57^ and Cys^146^. Moreover, the subunits fold to form eight-stranded, Greek-key *β* barrels, having 7 tethering loops, among which, loops IV (residues 49−83) and VII (residues 121−142), also known as the zinc and electrostatic loops, respectively, hold significant function. The zinc loop harbors all four Zn-interacting residues and a disulfide cysteine, Cys^57^. The electrostatic loop harbors a majority of the second-sphere active location residues, namely, the functionally significant Arg^143^, and serves as a gatekeeper that limits solvent access to the metal-interacting locations. SOD1 is generally activated by some posttranslational modifications like N-terminal acetylation, copper and zinc ion introduction, intramolecular disulfide bond generation between Cys^57^ and Cys^146^ [109], and dimerization (Figure 2).

### 4.3. Glutathione Peroxidase (GPX)

GPX belongs to a phylogenetically linked enzyme family that employs the reductant glutathione (GSH), which reduces H_2_O_2_ or organic hydroperoxide to water and alcohols, respectively (Table 1) [110]. The GPX catalytic center is a triad harboring Sec or Cys, Gln, Trp, and Asn [111]. However, in mammalian GPX8, the Gln is substituted by a Ser [112]. GPX1 was the first discovered selenoprotein [113], followed by GPX2~4 and GPX6, which were purified with a selenocysteine (Sec) in the catalytic center [114–118], and lastly, GPX5 and GPX7~8 with cysteine (Cys) [119–121].

GPXs serve an antioxidant role at various locations and cellular compartments. They are widely expressed within the cytosol, mitochondria, and colon (Figure 1) [122, 123], whereby they detoxify H_2_O_2_ and other soluble hydroperoxides [124–127]. Using this process, they protect erythrocytic hemoglobin from oxidative breakdown [128]. GPX2 is primarily found in the intestinal epithelium, and it is termed as GI-GPX or GPX-GI. In the intestine, it prevents the absorption of food-based hydroperoxides [129–131]. GPX3 is an extracellular enzyme produced by the proximal renal convoluted tubule [118], and it is basolaterally released into the plasma [132]. Several reports also identified GPX3 in other tissues like the basement membranes of intestinal epithelial cells [133]. GPX4, mainly located in the mitochondrial capsule of mature spermatozoa [134], facilitates reactions with more complex lipid hydroperoxides (LOOH) like phospholipid, cholesterol, and cholesterolester hydroperoxides, regardless of their location status [135]. GPX5 is an epididymal CysGPX in mice, rats, pigs, monkey, and humans, and it closely resembles GPX3. The GPX6 protein is yet to be purified, so its kinetic dynamics is undertermined. Thus, not much is known about it thus far. GPX7 is a CysGPX that strongly prefers the ER-related protein disulfide isomerase (PDI) as a reducing substrate [136]. This protein is either unexpressed or marginally expressed in breast cancer cell lines [121]. GPX8 is a CysGPX as well, and, like GPX7, it resides in the ER, where it partakes in oxidative protein folding [137, 138].

Based on the above evidences, the most significant GPXs are GPX1, GPX2, and GPX3. The GPX2 transcript is commonly expressed in all GI epithelial cells [130, 139]. However, the largest concentration is found in the ileum and cecum, which exhibits 2~3-fold elevation in GI-GPX transcript expression, compared to the remaining areas of rat GI [129]. GPX2 is uniformly available in the middle and lower portions of the GI [130]. The largest GPX2 protein concentrations occur in the colonic crypt bases, and it reduces gradually at the top of the crypts or villi [140]. GPX1, which is usually only marginally seen at crypt bases, was remarkably upregulated in the very same areas that express GPX2 [141]. Kidney GPX3 can transport and bind to the intestinal basement membrane. GPX3 interaction is particular and targeted toward certain cells. This indicates that cells regulate their basement membranes to expose GPX3 interacting locations, based on the need of GPX3 enzymatic activity [133, 142].

GPX activity modulation, particularly selenoproteins, depends on the availability of selenium (Figure 2). Scientists studying this process formed the selenoprotein stratification, suggesting that selenoproteins are not generally available with selenium, and in multiple cases, selenium is rate-limiting. Certain selenoproteins degrade fast, whereas others remain till severe deficiency occurs. As a result, selenoproteins that degrade quickly fall low in the stratification, and those with superior stability rank high. Among the various members of SecGPXs, GPX2 leads the hierarchy and then GPX4, GPX3, and GPX1[143]. To elucidate GPX expression modulation, one must recall the eukaryotic mechanism of selenoprotein biosynthesis [144]. Selenoprotein biosynthesis requires a special tRNA, termed as tRNA^(Ser)Sec^ [145] and a stem loop structure in the 3′ UTR, called Sec insertion sequence (SECIS) [146]. SECIS binding protein-2 (SBP2) interacts with a 3′ UTR SECIS element to recruit eEFsec, a Sec-specific elongation factor, and SECp43, a tRNA methylase, prior to delivering the entire complex to the ribosome [147]. The eukaryotic initiation factor 4a3 (eIF4a3) binds multiple selenoprotein transcripts to prevent SBP2 interaction and, subsequent, translation [148]. EIF4a3 is activated during selenium deficiency and directly interacts with SECIS elements [149].

### 4.4. Peroxiredoxin (PRDX)

PRDX is a member of a widely expressed peroxidase family that reduces a wide range of peroxides, such as H_2_O_2_, lipid peroxide, and peroxinitrite (Table 1). They are detected in numerous organisms like bacteria, plants, and mammals. PRDX enzymatic activity requires a thiol-containing intermediate thioredoxin to serve as a reducing cofactor. Based on various locations and functions within cells, the stratification system separates PRDX protein into PRDX1, PRDX2, PRDX3, PRDX4, PRDX5, and PRDX6, and all six categories express in the intestine as antioxidative enzymes [150–155]. PRDX triggers H_2_O_2_ and organic hydroperoxide reduction into water and alcohols, respectively. PRDX protects cells from OS via detoxification of peroxides, and it serves as a sensor of H_2_O_2_-based signaling [156–159]. PRDX1~2 participates in growth factors and TNF-*α* networks via modulation of intracellular H_2_O_2_ levels [160]. PRDX-4 modulates H_2_O_2_-mediated upregulation of cytosolic NF-*κ*B via regulation of I-*κ*B*α* phosphorylation [158].

The peroxide active site structure and sequence are highly conserved among all PRDX classes. It contains a conserved cysteine residue, designated as “peroxidatic,” Cys (C_P_) that serves as the location of peroxide oxidation [161]. Peroxides promote C_P_-SH oxidation to cysteine sulfonic acid (C_P_–SOH), which, in turn, interacts with a different cysteine residue, named “resolving” Cys (C_R_), to generate a disulfide, which undergoes subsequent reduction via an electron donor to end one catalytic cycle [162]. In terms of C_R_, PRDXs are stratified into typical 2-Cys, atypical 2-Cys, and 1-Cys PRDX subfamilies. PRDX1~4 is a member of the typical 2-Cys, PRDX5 aligns with atypical 2-Cys, and PRDX6 is with 1-Cys PRDX [163].

The overoxidation of PRDX1~2 and phosphorylation of PRDX 1~4 are known to decrease peroxiredoxin activity, which, in turn, regulates enzyme activity (Figure 2). The conserved Cys of PRDX1 and PRDX2 is H_2_O_2_-sensitive and corresponds to Cys^51^. The conserved Cys^51^–SH undergoes selective oxidization by H_2_O_2_ to form Cys–SOH, which, in turn, interacts with the Cys^172^–SH of another subunit to generate an intermolecular disulfide. However, the sulfur atoms belonging to Cys^51^ and Cys^172^ are located relatively far from one another (~13 Å), and, therefore, intermolecular disulfide formation between these residues takes considerable time. As a result, the Cys^51^–SOH intermediate is sometimes oxidized to Cys–SO_2_H, prior to formation of a disulfide (8, 12–14). Since sulfinic acid does not undergo reduction, Cys–SO_2_H-based overoxidation of PRDX enzymes remains catalytically inactive. The CDK- (cyclin-dependent kinases-) mediated phosphorylation of PRDX1, PRDX2, PRDX3, and PRDX4 at the conserved Thr^89^ residue reduces peroxidase activity of peroxiredoxins [164]. Furthermore, Thr^89^ phosphorylation abrogates enzymatic activity via disruption of the decameric conformation [165]. Multiple scientists suggested that the dimeric states of PRDX display reduced activity, compared to the decameric states [166–168]. Targeted PRDX C termini proteolysis suppresses peroxide-based inactivation, under high levels of peroxide. This is yet another proposed mechanism of peroxidase activity regulation [169].

### 4.5. Heme Oxygenase (HO)

HO is an oxidase enzyme with varied function. In the process of hemoglobin catabolism, with sufficient molecular O_2_ and reduced NADPH, HO promotes heme degradation to iron, carbon monoxide, and biliverdin. The two reported HO isoforms are HO-1 and HO-2, and they both express in the ER. HO-1 is a 32 kDa heat shock protein, and it is sensitive to a myriad of toxic stimuli, in locations, such as the lung [170, 171], liver [172, 173], and intestine [174–176]. Most inducers produce OS, including heme and heavy metals [177], hyperoxia [178], hypoxia [179], H_2_O_2_ [180], hyperthermia [181], and endotoxin [182]. HO-2 is a 36 kDa protein, and it is ubiquitously expressed in the brain and testis [183].

This review focuses on HO-1, which is mainly located in the intestine (Figure 1). HO-1 is a 288-residue protein [184, 185] composed mainly of *α*-helices, with heme in between the distal and proximal helices [186]. The conserved glycines associated with the distal helix facilitate flexibility, which causes the two HO molecules in the crystallographic unit cell to be varied. In one HO, the active pocket remains fairly open, with loose distal helix-heme interaction. The corresponding HO forms tighter heme-distal helix connection. This, along with the elevated crystallographic thermal values, indicates that the distal helix flexibility promotes the opening and closing of the heme pocket to facilitate interaction with the heme substrate and simultaneously allow dissociation of the biliverdin product. Similar to cytochromes P450, HO also oxidizes an unstimulated carbon center, whereas P450 employs a cysteine thiolate heme ligand that critically regulates O_2_ activation. HO is very similar to the globins in the O_2_-heme iron ligation, induced by an axial histidine ligand [187, 188].

HO-1 expression is modulated transcriptionally via porphyrins, metals, progesterone, other molecules, and it is expressed under OS, ischemia, hypoxia, and other disease conditions [189]. The human HO-1 5′ flanking region contains multiple regulatory elements [190], and thus far, transcriptional regulation appears to be the major form of HO-1 regulation by most, if not all, agents. HO-1 research currently focuses on the recognition and characterization of cis-acting DNA elements and their cognate binding proteins that carry out activation of gene transcription. In a majority of cases, these motifs are similar to or slightly differ from recognition sites for reported DNA-interacting proteins, namely, Fos/Jun (AP-l) and NF-*κ*B/Rel protein family, which are two primary OS-inducible TFs in mammalian cells [191].

## 5. Intestinal Reactive Species (RS)

Of note, the terminology ROS should be appropriately named using the particular chemical species under consideration. O_2_ often reduces to free radicals (e.g., free electron like the O^2•−^ anion radical and the hydroxyl radical). In contrast, H_2_O_2_ is the product of two-electron reduction. Since it is not a radical, it is chemically stable. Electronic excitation often includes a single molecular O_2_ and excited carbonyl compounds (i.e., nonradicals). Additional physiologically significant RS include chlorine and bromine species like hypohalous acids, hypochlorite, and chloramines. Free radicals, generated under both normal and pathological conditions, are extremely reactive and have an unsatisfied electron valence pair. These are very damaging to tissues, as is reported in cases of radiation, environmental chemical damage, and aging. Often, “free radicals” and “ROS” are utilized interchangeably. This may be correct in most cases, but, in certain instances, this terminology can be misleading.

O_2_^·—^ is the most significant ROS, and it can produce other reactive O_2_ intermediates. The inner mitochondrial membrane (IM) harbors a multitude of enzyme complexes commonly known as the mitochondrial respiratory chain (MRC). The MRC contains complexes I-IV (NADH-ubiquinone oxidoreductase, succinate dehydrogenase, ubiquinol-cytochrome c oxidoreductase, and cytochrome c oxidase (CCO)), coenzyme Q (CoQ), and a peripheral protein cytochrome c located on the surface of the IM. MRC complexes I and III release elections that reduce molecular O_2_ to form O_2_^·—^[192]. CCO (complex IV) is the last MRC-associated enzyme to reduce O_2_ to two H_2_O molecules using a four-electron reduction [193]. Complex IV is not regarded as a physiologically significant ROS origin [194]. Instead, it was shown to serve as a mitochondrial antioxidant that oxidizes O_2_^·—^to O_2_ [195]. In case of elevated cellular O_2_, CCO is oxidized and uses ^·^NO. But, under reduced O_2_ levels, NO accumulates within the cell [196].

Indeed, not all free radicals induce OS. In fact, OS is intricately linked to ROS. ROS is extremely reactive, and it is constantly generated via cellular respiration and various enzyme reactions. Among common ROS are as follows: O_2_^­˙^, hydroxyl radicals (˙HO), LOOHs, and reactive nonradical compounds like singlet O_2_ (^1^O_2_), H_2_O_2_,and hypochlorous acid (HOCl) [197]. RNS includes radical compounds like ˙NO, nitrogen dioxide (˙NO_2_), and nonradical compounds like peroxynitrite (ONOO­) and dinitrogen trioxide (N_2_O_3_).

A majority of the above compounds have poor stability due to the unpaired electrons in their outer electron orbit. Upon ROS accumulation, chief cellular antioxidants like GSH and thioredoxin undergo altered redox states, thereby decreasing antioxidant defenses. Therefore, based on the above review of intestinal oxidoreductases, RS are classified in Table 3. RS are crucial for the maintenance of intestinal physiological activities, and this review will detail the significance of RS in intestinal OS.

## 6. The “Tai Chi” Theory of Intestinal Redox System

Oxidative stress is an imbalance between oxidants and antioxidants in favor of oxidants that leads to the disruption of redox signaling and/or molecular damage [198]. OS stratification, based on intensity, is as follows: basal, low, intermediate, and high intensity OS are generally abbreviated as BOS, LOS, IOS, and HOS [199]. BOS also represents physiological OS or oxidative eustress (OeS) [200]. Pathological OS is often termed as OS, but the limitation between OeS and OS is not clear. In fact, cellular H_2_O_2_ concentration of 0.1 *μ*M can be considered as OeS or OS in different cellular states [201].

Under BOS conditions, OS is so negligible that it cannot be measured using traditional approaches. Augmented ROS dosages can trigger LOS, in which case, both oxidatively modified structures and endpoint parameters like ROS-driven ROS-sensitive parameters can be measured. LOS may be divided into two components, increasing and decreasing, after it passes maximum. Upon further dosage increases, cells enter the IOS and then HOS stage. In the HOS, both measured function plateau, meaning that all available substrates become potentially oxidized, thus achieving a near maximum response. The redox enzyme regulation is critical for the above complex processes. The specific modulation of each enzyme was described in the previous paragraph, and the regulation mechanism of redox enzyme is summarized below.

### 6.1. The Thiol/Disulfide in Intestinal Redox Regulation

Under normal conditions, extracellular GSH concentrations remain quite scarce, other than in the intestinal lumen (60–300 *μ*M), where GSH levels are high, due to elevated levels in the bile (1–2 mM in rat bile) [202] and dietary intake [203]. Luminal GSH catalyzes dietary disulfide reduction, peroxidized lipid metabolism, xenobiotic detoxification, and mucin oligomer assembly to maintain mucus fluidity [202, 204, 205]. Daily dietary lipid peroxide intake can reach around 1.4 mmol, with an 84 g fat intake [206]. Luminal and intracellular GSH strongly safeguards against dietary lipid peroxides [207]. In rats chronically fed with lipid peroxides, GSH supplementation protects against lipid peroxide-mediated suppression of mucosal proliferation [208]. Increasingly, scientists believe that in biological systems, the GSH/GSSG redox, in combination with Trx/TrxSS and Cys/CySS, forms distinct redox modulation nodes that regulate cell metabolism and growth [209]. Given that all redox couples exist without equilibrium, their function as an on-off sulfur switch supports the distinct modulation of a singular protein or protein sets during normal cellular function [210].

The Cys/CySS redox couple, with partial help from the GSH system, modulates the extracellular/luminal redox environment [211]. The plasma Cys/CySS and GSH/GSSG redox couples become displaced from equilibrium carrying Eh values strongly set at −80 mV and −140 mV, respectively [209, 212]. The true extracellular Cys and CySS concentrations are relatively low, at 40 *μ*M and 8-10 *μ*M, respectively, and are determined based on the Cys/CySS in the diet [213], GSH hydrolysis [214], thiol-disulfide exchange reactions [215], and Cys/CySS shuttle [216]. An oxidized plasma Cys/CySS redox state is strongly correlated with vascular diseases, such as diabetes, cardiovascular disease, and atherosclerosis [212]. Therefore, plasma Cys/CySS alterations may predict health and disease [217]. Luminal Cys/CySS maintains the thiol-disulfide redox states of extracellular proteins [218] and lumen [219]. In rat intestine, GSH hydrolysis, necessary for nutrient absorption [220] and mucus preservation[221], produces ~40% luminal Cys. The luminal thiol-disulfide redox status is modulated via the Cys/CySS shuttle [211] and includes luminal Cys export [219], GSSG reduction, and CySS synthesis [211], with subsequent CySS absorption [222], intracellular GSH-based CySS reduction, and Cys resecretion into the lumen. In polarized Caco-2 cells, Cys/CySS Eh at basal and apical surfaces are modulated at varying rates [223], suggesting stand-alone redox networks at corresponding polar membrane surfaces.

### 6.2. H_2_O_2_ in Intestinal Redox Regulation

H_2_O_2_ does not have charge and is, therefore, optimal for redox sensing and signaling [224, 225]. Despite being known for extremely slow reactions with biomolecules (second-order rate constants approximately 1/M/s), H_2_O_2_ reacts well with certain residues, for instance, some cysteinyl residues in peroxiredoxins or selenocysteinyl residues in GPXs (10^7^/M/s) [226]. Also, since H_2_O_2_ has a sluggish reaction, it is able to diffuse further from the production site to react with targets some distance away. In contrast, highly reactive oxidants like hydroxyl radical exhibit a more localized reaction.

Multiple studies suggested potential roles of thiol peroxidases as H_2_O_2_ sensors and transducers. H_2_O_2_ is a crucial member of redox networks [227]. For instance, peroxiredoxin-2 is a highly sensitive primary H_2_O_2_ receptor that particularly conveys oxidative equivalents to the redox-modulated TF STAT3 to generate a redox relay for the H_2_O_2_ redox network [228]. Another way of spatiotemporal modulation is the H_2_O_2_-mediated hyperoxidation of peroxiredoxin cysteinyl residues to sulfinic acid. This, in turn, inactivates the peroxidase. This event produces a large buildup of H_2_O_2_ at target sites, which then facilitates oxidation of targeted proteins [229]. Sulfiredoxin reduces the hyperoxidized peroxiredoxins, thus closing the functional loop and restoring functionality [230]. Hence, when confined close to the physiological H_2_O_2_ concentration range (i.e., 10 nM) [231, 232], H_2_O_2_ acts as an appropriate secondary messenger in redox signaling. Based on the conclusions of two studies, an intact liver H_2_O_2_ production rate is around 50 nmol H_2_O_2_ per min per gram, which is about 2% of the entire O_2_ uptake under steady conditions [232, 233].

### 6.3. The “Tai Chi” Theory Based on Intestinal Redox Regulation

There are many theories aimed at generalizing the function and regulation of the intestinal redox system, such as the redox species balance regulated by the intestinal oxidoreductases involves in various physiological functions including absorption and defense [234, 235]. But this “balance” cannot accurately describe the operational mode of the intestinal redox system. Firstly, each oxidoreductase primarily mediates its action via translational or posttranslational processing, which does not necessarily maintain the balance of enzymatic activities. Additionally, the way of the oxidoreductase products, particularly thiol/disulfide or H_2_O_2_, maintaining the redox balance is not simply by downregulating or upregulating its level or activity, otherwise known as “dynamic balance” or “homeostasis” which is more appropriate. Moreover, given the above factors, at cellular, tissue, and whole level, the “balance” alone cannot appropriately explain the mechanism of the intestinal redox system under physiological or pathological status.

**Figure 3:**
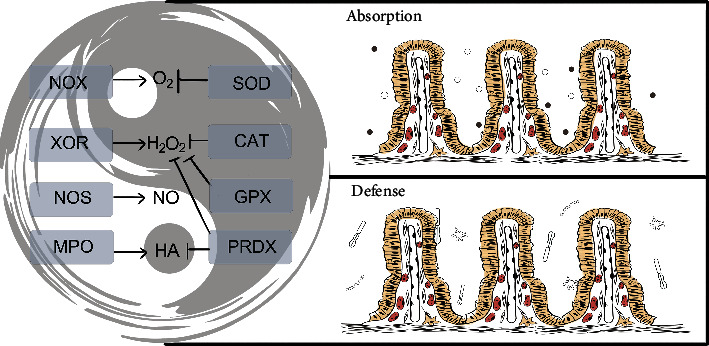
The intestinal oxidation-reduction (redox) system “Tai Chi” in normal physiological function. To support normal physiological function, the level of redox species was maintained in a certain limitation using subtle and complex regulatory methods, including local (induced by the oxidoreductases in different cell, tissue, and organ location) and global (induced by the products of oxidoreductases inside and outside the cell, even inside and outside the intestine) horizontal regulation. Additionally, the intestinal physiological function mainly includes the absorption of nutrients (water with the white point and inorganic salt with the black point) and the defense against pathogenic microorganisms.

Therefore, with reference to the concept of ancient Chinese medicinal theory, we proposed the “Tai Chi” theory of the intestinal redox system to explain the intestinal redox system (Figures 2 and 3). The “balance” theory is inexact that the level of redox species maintains the intestinal redox balance via a “down or up” regulation. The intestinal redox system supports the physiological function by a subtle and complex regulatory ways, including local (induced by the oxidoreductases in different cell, tissue, and organ locations) and global (induced by the products of oxidoreductases inside and outside the cell, even inside and outside the intestine) horizontal regulation. We summarized the regulation and function of redox system as the “Tai Chi” theory for its mutually reinforced and neutralized elements and maintenance of homeostasis with a certain amount of redox species.

## 7. Overview of The Intestinal RedoxSystem in CIM

**Figure 4:**
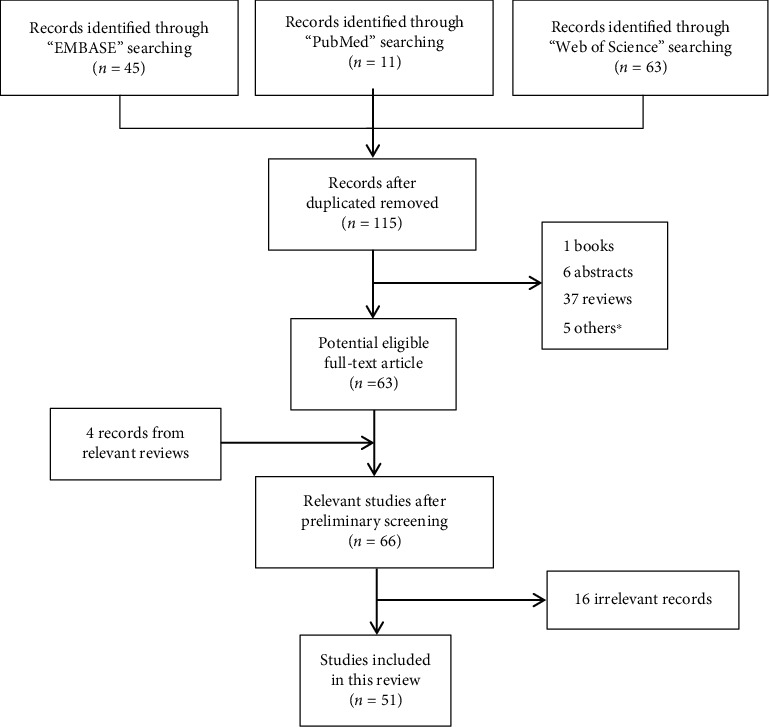
A flow diagram of the identification of articles included in this review.

Our article selection process is detailed in supplemental information. Based on the module and search string in EMBASE (Table S1), PubMed (Table S2), and Web of Science (Table S3), we established the retrieval strategy of the above respective databases (Table S4). Overall, 51 articles were selected from the date of inception till July 31, 2021 (Figure 4). Using literature analysis, we revealed that ROS was critical for CIM pathology, and it was induced by only five chemotherapeutic drugs, namely, methotrexate (MTX), 5-fluorouracil (5-FU), cisplatin, irinotecan (CPT-11), and oxaliplatin.

The existing literature and mainstream academic view is that ROS is produced rapidly in the initial stage as the chemotherapy drugs cause direct damage to intestinal mucosa cells, thus resulting in necrosis, mitochondrial dysfunction, and further ROS generation. Through our review of the intestinal redox system, we revealed that the intestinal redox enzyme system is very complex and is regulated by multiple factors. Thus, ROS production is not limited to the early stage but is present throughout the entire course of CIM. Therefore, the redox system is involved in all stages of CIM.

Given that some chemotherapeutic drugs produce ROS using different redox enzymes, we first elaborated the mechanism of oxidoreductase and its modulation by chemotherapeutic drugs, in this review. In our analysis, we primarily selected articles that examined alterations in the redox enzyme level or activity as indicators of CIM severity. To circumvent limitations based on our literature selection, we also explored the structural and regulation characteristics of intestinal oxidoreductase to further elucidate their significance in CIM pathogenesis.

## 8. Oxidoreductases in CIM

### 8.1. 5-Fluorouracil (5-FU)

(5-) FU is a highly prevalent chemotherapeutic medication for the management of multiple forms of cancer [236]. Unfortunately, approximately 50–80% 5-FU consumers eventually develop mucositis and discontinue chemotherapy [237, 238]. 5-FU also causes diarrhea likely via a multifactorial network involving acute harm to the mucosal intestine (such as intestinal epithelial loss, superficial necrosis, and inflamed bowel wall), thereby causing an imbalance between absorption and secretion within the small intestine [239]. In a retrospective study, it was observed that 5-FU elevates NOS and MPO, while reducing CAT, SOD, GPX, PRDX, and HO (Table 4). This, in turn, enhances O^2•−^, H_2_O_2_, lipid peroxides, nitrate, and protein carbonyls, while diminishing GSH and NO. 5-FU also triggers an excessive NF-*κ*B production, which modulates oxidoreductases to regulate CIM progression [240–243]. In 5-FU-mediated CIM, enteric glial cells secrete S100B to activate neuronal NF-*κ*B axis in a RAGE-reliant fashion. This triggers glial cell to secrete iNOS-based NO and OS [240]. Other researchers reported that 5-FU enhances NF-*κ*B levels [241–243], which is the sole basis of the CIM-based oxidoreductases regulation. There is conclusive evidence that MPO (via TLR-4/NF-*κ*B) [244], HO (NF-*κ*B/Rel) [191], and CAT (AKT/NF-*κ*B) [84, 245] regulation occurs via the intestinal NF-*κ*B-related pathway.

Another significant mechanism underlying 5-FU-based CIM is the Nrf pathway-mediated regulation of HO [246, 247]. 5-FU activates NF-E2-related factor 2 (Nrf2), which decouples from the actin-related Keap1 protein to transfer to the nucleus, whereby it promotes HO-1 cytoprotective gene transcription [248, 249]. However, the mechanism of ROS–scavenging oxidoreductase HO-1 in CIM is controversial. HO-1 was shown to be either up- [247] or downregulated[250] in 5-FU-based CIM, and this may be due to a compensatory mechanism. In normal physiological conditions, cells balance excessive ROS production with ROS elimination via scavenging systems like intracellular redox-balancing gene HO-1, phase II detoxifying gene quinone oxidoreductase-1 (NQO-1), and genes encoding transporters (multidrug-resistant proteins) [251]. A majority of the above genes possess an enhancer sequence termed as the antioxidant response element [252–254], which is activated by the TF Nrf2. Considering the physiological roles of these are-containing genes, it is possible that Nrf2 target gene activation will likely enhance detoxication of xenobiotics like chemotherapeutic drugs, in order to protect cells from ROS-induced apoptosis [255].

5-FU is highly efficacious in boosting CIM-induced apoptosis. This is carried out via downregulation of Bcl-2 expression while upregulating Bax levels [242, 250, 256–258]. Apoptosis is a systematic process involving multiple genes like caspase-3, Bcl-2, and Bax. Bcl-2 strongly modulates apoptotic signaling and is, therefore, referred to as a survival-promoting protein. In contrast, Bax is regarded as the opposite of Bcl-2; thus, it is a proapoptotic protein [259]. Additionally, suppressing the ATF4/Chop/Bcl-2/Bax network inhibits the activation of SOD, GPX, and CAT. In ATF4-KD mice, for example, both intestinal GPX and CAT levels were markedly elevated, relative to AFT-WT mice [260].

### 8.2. Methotrexate (MTX)

MTX is a folic acid counterpart that suppresses dihydrofolate reductase enzyme activity. It is a highly efficacious chemotherapeutic drug against acute leukemia, trophoblastic disease, and intraosseous sarcoma, and it was recently employed as an antirheumatic drug [254]. MTX induces GI toxicity that manifests as diarrhea, nausea, and reduced nutrient absorption. CIM is highly prevalent in MTX therapy. The MTX-induced CIM causes villus atrophy or crypt loss, as evidenced by histology [261–264]. Based on a retrospective investigation, MTX enhances xanthine dehydrogenase and MPO, while diminishing CAT, SOD, and GPX activity (Table 4). This results in the augmentation of peroxynitrite, lipid peroxides, nitrate, and protein carbonyls, while reducing GSH activity.

MTX also induces mitochondrial dysfunction and respiratory chain defects during CIM [265–267]. It triggers enterocytic ultraconformational alterations like swollen mitochondria, excessive crista disruption, altered mitochondrial morphology, ER and GA dilatation/cytoplasmic vacuolation, and microvilli degeneration and fragmentation. In a study examining MTX effects on treated cells, the mitochondrial damage was obvious after only 6 h, and the severity of damage only increased with time. The mitochondria were swollen after 6 h of MTX therapy, and cristae were disrupted after 12 h. The crista disruption was more extensive at 24 h, with complete crista dissolution at 48 h. The ER and GA dilatations were regarded as degenerative vacuolar appearance at 24 h following MTX therapy. Based on the Trump-defined classification of mitochondrial injury (MI), the enterocyte MI score of MTX-administered rats was >4, suggesting considerable MI that can initiate the apoptotic and/or necrotic process in enterocytes. MTX drastically decreases (>70%) ETC complex II and IV activities, without any change to complex I and III activities. Hence, it is not difficult to understand that the function of redox enzymes, including SOD and GPX, is primarily located within the mitochondria, and it decreases with mitochondrial dysfunction.

MTX strongly upregulates TNF-*α* transcript and protein expressions during CIM [268–270]. In turn, TNF-*α* induces XDH activity in renal epithelial cells [271] and RAW 264.7[272]. Moreover, human XDH, which is ubiquitous in the liver and intestine, is modulated by multiple promoter element-binding factors [40]. To identify relevant human XDH gene 5′-flanking region promoter elements, one study examined the 200 base pair sequence of the 5′-flanking region. The scientists identified candidate docking regions for factors associated with inflammation and acute phase response. In particular, they demonstrated four CCAAT/enhancer protein interacting sites, three IL-6 RE, one NF-*κ*B site, possible TNF-*α*, interferon-*γ*, and interleukin-1 RE. Regrettably, even though we can infer that the activity of XDH, ROS, and TNF is increased in MTX-induced CIM, the direct or indirect relationship between them still remains under investigation.

MTX enhances PARP levels in the duodenum and ileum [273], with the villi and crypts exhibiting high intensity focal expression. In contrast, the jejunum rarely exhibited this level of expression. The Alu receptor RE (AluRRE) that is upstream of the MPO gene is a standard member of the principal Alu subcategory, and it carries four hexamer half sites linked to the consensus AGGTCA, and it is identified by members of the nuclear receptor superfamily of ligand-dependent TFs [79, 80, 274]. The hexamers form direct repeats with spacings of 2, 4, and 2 bp [275]. PPARc–RXR heterodimers interact with the third and fourth hexamers, and the PPARc ligands markedly accelerate human MPO production in MCSF-M/(~20-fold), while inhibiting MPO in GMCSF-M/(~20-fold) [80]. Nevertheless, MTX increases MPO, but whether this involves PARP activation remains to be seen.

MTX also interacts with histone to form an MTX-protein complex that diminishes histone acetyltransferases (HAT) activity [276]. MTX was shown to bind Lys^9^ on the histone stick model and the Lys^37^, and Arg^40^-associated histones showed the strongest binding between medication and protein. Additionally, the protein complex-based MTX-driven reduction in histone acetylation pattern also reduces HAT activity. Furthermore, acetylation on histone induces higher CAT mRNA expression [277], which, in a feedback look, induces MTX-driven downregulation of CAT via acetylation.

### 8.3. Others

Cisplatin is highly efficacious and, therefore, commonly employed in cancer therapy. However, it is known to elicit numerous adverse effects [278–280], including CIM [281, 282]. The structural CIM presentations include loss of crypt and villus atrophy. Functionally, CIM presents as impairments in absorption and barrier integrity [281, 283]. A retrospective analysis revealed that cisplatin and oxaliplatin drastically increased NOS, while decreasing CAT, SOD, and GPX. This, in turn, enhanced NO, lipid peroxides, and protein carbonyls, while reducing GSH levels. Similar to camptothecin, irinotecan hydrochloride (CPT-11) is an antiproliferative medication used to manage multiple forms of solid tumors. But, CPT-11 is also associated with adverse effects like CIM [284]. The CPT-11-induced intestinal damage is characterized by enhanced jejunal crypt apoptosis and destruction of colon villi, while decreasing goblet cells. Owing to these changes, patients often experience bloating, abdominal pain, diarrhea, and weight loss. Based on our retrospective analysis of relevant literature, CPT-11 upregulates NOX and MPO, while downregulating CAT, SOD, GPX, and HO, which results in increased levels of NO, H_2_O_2_, and lipid peroxides, nitrate, while decreasing levels of GSH and NO (Table 4). Similar to 5-FU, CPT-11 downregulates HO via the Keap-1/Nrf-2 pathway [285]. The TF Nrf2 is a critical redox switch that modulates levels of antioxidant and protective enzymes. Moreover, the enzyme activities and sensitivities are modulated by the ubiquitin ligase adaptor Kelch-like ECH-associated protein- (Keap-) 1[286]. Finally, it is unique that CPT-11 decreases HO expression thus inactivating the Keap-1/Nrf-2 pathway.

## 9. RS in CIM

The core of the pathological mechanism of CIM is the cascade amplification of inflammatory factors. Therefore, some scholars classified this as a special type of intestinal inflammatory disease and provided theoretical evidences and clues to further examine the networks related to RS in CIM.

### 9.1. The Role of RS in CIM-Based Inflammation

**Figure 5:**
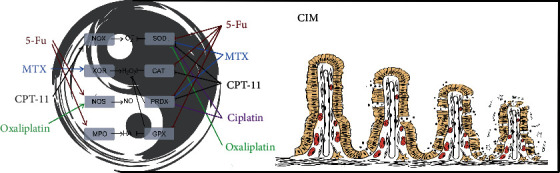
The intestinal oxidation-reduction (redox) “Tai Chi” system in CIM pathogenesis. MTX, 5-FU, cisplatin, CPT-11, and oxaliplatin induced high levels of O_2_^−^, H_2_O_2_, NO, and HA via regulation of oxidoreductases, which disrupted the balance of intestinal redox “Tai Chi” system, and resulted in apoptosis and inflammation of the intestine, which contributed to the pathogenesis of CIM.

Five chemotherapeutic drugs induce ROS activation. Elevated ROS levels phosphorylate I-*κ*B*α* to initiate destruction of the protein, thereby releasing NF-*κ*B, which then relocates to the nucleus [287]. This ROS/NF-*κ*B self-sustaining modulatory network likely contributes to the continuation and aggravation of chronic inflammation [288, 289]. NF-*κ*B activation triggers the expression of some 200 genes, a majority of which regulate mucosal toxicity [288]. Genetic activation via chemotherapy-triggered TF activation was shown to augment proinflammatory cytokines TNF-*α*, IL-1*β*, and IL-6 production [4]. Moreover, these proteins accumulate in the mucosa. The increase in proinflammatory cytokines likely initiates the early destruction of connective tissue and endothelium, thereby enhancing mesenchymal–epithelial signaling, while decreasing epithelial oxygenation and, eventually, developing epithelial basal-cell death and injury (Figure 5).

### 9.2. The RS-Mediated Apoptosis in CIM

NO triggers intestinal apoptosis, which contributes to CIM pathology. It was reported that enhanced iNOS activity and immunoreactivity to 3-nitrotyrosine coincided perfectly with areas of intestinal necrosis or apoptosis. Hence, it is very likely that NO causes enterocytic apoptosis [290]. Multiple reports indicate that the mitochondria are critical for cell apoptosis. Mitochondrial cytochrome c secretions, along with the membrane depolarization, dominate the early stages of apoptosis. Furthermore, cytochrome c directly activates caspase-3 [290]. Richter [291] revealed that a reduction in cellular adenosine triphosphate (ATP) concentration triggers apoptosis. However, maintaining the mitochondrial membrane potential (i.e., the driving force for ATP production) prevents apoptosis, and dysregulating it releases proapoptotic factors. There is evidence that NO can directly alter mitochondrial membrane permeability to promote apoptosis. Moreover, the NO effects were abrogated using specific inhibitors of mitochondrial permeability transition [292].

LOOH is yet another RS that triggers apoptosis of human colonic CaCo-2 cells using redox shifts [293]. Using even subtoxic LOOH concentrations enhances CaCo-2 cell apoptosis. This LOOH-triggered apoptosis strongly correlates with marked reductions in the GSH-to-oxidized glutathione (GSH/GSSG) ratio, which typically precedes DNA fragmentation. GSH oxidation by the thiol oxidant diamide markedly diminishes cellular GSH, as well as the GSH/GSSG ratio, and it correlates with caspase-3 activation and PARP cleavage. This confirms a temporal relationship between cellular redox imbalance and apoptotic cellular death. These kinetic investigations further revealed that the oxidant-mediated early redox alteration is a primary inciting event in the apoptotic cascade. Once initiated, the redox balance recovery fails to protect against apoptosis of CaCo-2 cells. Taken together, the subtoxic LOOH levels dysregulate intestinal redox homeostasis, and this eventually leads to cell apoptosis.

## 10. The Intestinal Redox Regulation in CIM

It is interesting that the GSH/GSSG balance is a critical factor in the modulation of the intestinal redox system, and it is also involved in the intestinal apoptotic pathway [293]. An alteration of the GSH/GSSG ratio is intricately linked to caspase-3 activation. This strongly correlates dysregulated redox homeostasis to apoptosis initiation. Scientists observed a comparable relationship between GSH oxidation and apoptosis in fibroblast cells, upon serum withdrawal. Moreover, the BOS-induced loss of cellular GSH, without alteration to the GSH/GSSG ratio, displayed no initiation of cell apoptosis. Instead, cell apoptosis was closely related to enhanced GSSG, and not GSH. Furthermore, multiple studies indicate an influence of H_2_O_2_ in intestinal apoptosis as well [294, 295].

## 11. The Intestinal Redox System and Its Role in CIM Treatment Strategies

There are 39 treatment strategies from the studies included in this review. These strategies can be further divided into 7 classes: traditional Chinese medicine (TCM), nature products, synthesis compound, food extract, probiotics, vitamin, amino acid, and others (Table 5). Unfortunately, all studies used animal models or cells as research objects and were not clinical studies. This also indicates that these treatment strategies have certain limitations. Most of them regard the intestinal redox system as the effect indicator of CIM, without in-depth discussion of the mechanism whereby the redox reaction is influenced and plays a role in the treatment of CIM.

**Figure 6:**
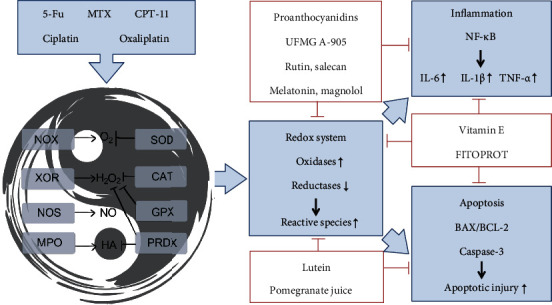
The mechanism of CIM treatment strategies in studies included in this review. The treatment strategies can be divided into 3 categories, according to the targets: proanthocyanidins, UFMG A-905, rutin, salecan, melatonin, and magnolol target redox and inflammation; lutein and pomegranate juice target redox and apoptosis; and vitamin E and FITOPROT target redox, inflammation, and apoptosis.

Based on our review of relevant literature discussing the mechanism of various CIM treatment strategies, these treatment strategies can be divided into 3 categories, according to the targets: proanthocyanidins, UFMG A-905, rutin, salecan, melatonin, and magnolol target redox and inflammation; lutein and pomegranate juice target redox and apoptosis; and lastly, vitamin E and FITOPROT target redox, inflammation, and apoptosis (Figure 6). As we discussed earlier, NF-*κ*B (in inflammation) and caspase-3 (in apoptosis) are the key target molecules influenced by the intestinal redox system, and the above treatments relieve CIM mainly via NF-*κ*B and caspase-3 regulation. However, it is worth noting that the experimental evidence of the studies is not sufficient, particularly in terms of the validation of the pathway, and there is a lack of omics research as well. It is encouraging that there are more and more studies involving TCM, which also applies our “Tai Chi” theory to elaborate the intestinal redox system and its role in the short-term treatment strategies involving CIM.

## 12. Conclusion

Based on the elaboration of the intestinal redox enzyme and its regulation mechanism, considering that the regulation of the OS level is not simply described as “increased” or “decreased,” and it is observed both inside and outside the cell, the intestine, and even the body, we preliminarily proposed the intestinal redox “Tai Chi” theory and attempted to reveal the role of OS events in the pathogenesis of CIM. However, current studies involving CIM employed oxidoreductases and reactive species as evaluation index of disease, which is inappropriate, as per our review. Moreover, the intestinal redox system participates in the pathogenesis and affects the prognosis of CIM, which is still worthy of in depth study.

## Figures and Tables

**Table 1:** Major oxidoreductive enzymatic reactions of oxidoreductases in intestine.

Enzyme name	Enzymatic reaction	Cofactor
NADPH oxidase	NADPH+2 O_2_=2 O_2_^.-^+NADP^+^ +H^+^	
Xanthine dehydrogenase	H_2_O+xanthine+O_2_=H_2_O_2_+urate	[2Fe-2S] cluster, FAD, Mo-molybdopterin
Nitric oxide synthase	H^+^+4 O_2_+3 NADPH+2 L-arginine=4 H_2_O+L-citrulline+3 NADP^+^+ 2NO	Heme, FAD, FMN, tetrahydrobiopterin (BH4)
Myeloperoxidase	Chloride+H^+^+H_2_O_2_=H_2_O+hypochlorous acid	Ca^2+^, Heme *b*
Catalase	2H_2_O_2_=2H_2_O+O_2_	Heme, NADP^+^
Superoxide dismutase		
SOD1	2H^+^+2 O_2_^.-^=H_2_O_2_+O_2_	Zn^2+^, Cu^+^
SOD2		Mn^2+^
SOD3		Zn^2+^, Cu^+^
Glutathione peroxidase		
GPX1~3, 5~8	2 glutathione+H_2_O_2_=glutathione-2S+2 H_2_O	
GPX4	1 hydroperoxy polyunsaturated fatty acid+2 glutathione=1 hydroxy polyunsaturated fatty acid+glutathione-2S+H_2_O	
Peroxiredoxin		
PRDX-1~5	[thioredoxin]-dithiol+1 hydroperoxide=[Thioredoxin]-2S+1 alcohol+H_2_O	
PRDX-6	1 hydroperoxide+2 glutathione=1 alcohol+glutathione-2S+H_2_O	
Heme oxygensae		
HO-1	Heme *b*+3 O_2_+3 reduced [NADPH-hemoprotein reductase]=Biliverdin IX*α*+CO+Fe^2+^+H^+^+3 H_2_O+3 oxidized [NADPH-hemoprotein reductase]	
HO-2		

**Table 2:** Subcellular location of oxidoreductases in intestine.

Enaymes name	Cytoplasm	Melanosome	Endosome	Endoplasmic reticulum	Mitochondrion	Peroxisome	Lysosome	Extracellular	Plasma membrane	Nucleus	Golgi	Cytoskeleton	Cell junction
NADPH oxidase													
NOX1									√				
NOX2									√				
NOX3													
NOX4				√					√				√
DUOX1									√				
DUOX2									√				√
Xanthine dehydrogenase	√					√		√					
Nitric oxide synthase													
NOS1									√				
NOS2	√												
NOS3									√		√	√	
Myeloperoxidase							√						
Catalase						√							
Superoxide dismutase													
SOD1	√				√					√			
SOD2					√								
SOD3								√			√		
Glutathione peroxidase													
GPX1	√				√								
GPX2	√												
GPX3								√					
GPX4	√				√								
GPX5~7								√					
GPX8									√				
Peroxiredoxin													
PRDX1	√	√											
PRDX2	√		√										
PRDX3	√			√	√								
PRDX4	√												
PRDX5	√				√	√							
PRDX6	√						√						
Heme oxygensae													
HO-1				√									
HO-2				√									

**Table 3:** Reactive species in intestine.

	Free radicals	Nonradicals
Reactive oxygen species	Hydroxyl radical	Hydrogen peroxide
	Superoxides	Hypochlorous acid
	Singlet oxygen	Lipid peroxides
		Prostaglandin endoperoxides
		Electronically excited carbonyls
Reactive nitrogen species	Nitric oxide	Nitrate
		Peroxynitrite

**Table 4:** Oxidoreductases and redox species in CIM.

	5-FU	MTX	CPT-11	Cisplatin	Oxaliplatin
Oxidoreductases					
NADPH oxidase			↑[297]		
Xanthine dehydrogenase		↑[298]			
Nitric oxide synthase	↑[240, 241, 250, 299]				↑[300]
Myeloperoxidase	↑[241, 246, 257, 258, 299, 301–309]	↑[269, 298, 310]	↑[285, 297, 311–313]		
Catalase	↓[239, 242, 247, 304, 314–316]	↓[263, 310]	↑[312]		
Superoxide dismutase	↓[239, 242, 246, 247, 257, 304, 317]	↓[263, 298, 310, 318]	↓[285]	↓[319, 320]	↓[300]
Glutathione peroxidase	↓[242, 257, 310]	↓[263, 310, 318]	↓[285]	↓[319, 320]	
Peroxiredoxin	↓[265]				
Heme oxygenase	↓[250] ↑[247]		↓[285]		
Redox species					
ROS	↑[242, 243, 246, 250, 256, 321]	↑[263, 298, 322, 323]	↑[311, 312]		
Superoxides	↑[246]				
Nitric oxide	↑[247, 257]		↑[285]		↑[300]
Hydrogen peroxide	↑[302, 304]		↑[324]		
Peroxynitrite		↑			
Lipid peroxides	↑[239, 241, 247, 257, 258, 301–304, 308, 314, 315, 317, 325, 326]	↑[263, 269, 270, 310, 318]	↑[285, 297, 311–313, 325]	↑[319, 320, 327]	↑[300]
Nitrate	↑[240, 308, 314]	↑[273]	↑[312]		
Protein carbonyls	↑[239]	↑[310]		↑[327]	
GSH	↓[242, 247, 257, 301, 303, 307, 314]	↓[269, 270, 298]	↓[297, 311–313, 325]	↓[319, 320, 327]	↓[300]

**Table 5:** The treatment strategies to CIM in studies included in the review.

	Traditional Chinese medicine	Nature products	Synthesis compound	Food extract	Probiotics	Vitamin	Amino acid	Others
5-FU	Carboxymethyl pachyman [242] Saikosaponin-A [247] Aquilariae Lignum Resinatum [317] Rutin [301] Troxerutin [307] Taurine	FITOPROT [243] Diadzein [314] Cashew Gum [303] Proanthocyanidins [256] Ursodeoxycholic acid	Apolipoprotein mimetic peptide [299] MS-SOD [246]	Pomegranate Juice [250] Aḉaḯ	Fructo-oligosaccharides Lactobacillus UFMG A-905 [328] Exopolysaccharide [257]	Vitamin C Vitamin E [241]	L-Arginine L-Citrulline	
MTX	Lutein Rehmannia glutinosa Libosch [270]	Salecan [263]	Anakinra [269] Prostaglandin E [323]	Royal jelly [264]				Melatonin [273] Ozone
CPT-11	Gegen Qinlian decoction [285] Luteolin [312]	FITOPROT [313] Proanthocyanidins	Nanocomposite Fullerol [311]	Coffee ingredients	UFMG A-905			
Cisplatin			Se@Albumin nanoparticles [319]			Vitamin	D-Methionine	
Oxaliplatin		Magnolol						

## Data Availability

The data used to support this study are included within the article.

## Supplementary Materials

Supplementary Materials Using a similar modular search strategy described in Ranna et al. [296], the article selection process of the review is detailed in supplemental material.
