# Supplementary material for: The Intestinal Redox System and Its Significance in Chemotherapy-Induced Intestinal Mucositis
Source: Oxid Med Cell Longev. 2022 May 9;2022:7255497. doi: 10.1155/2022/7255497 (PMC9110227; doi:10.1155/2022/7255497)
Supplement: Supplementary Materials — Using a similar modular search strategy described in Ranna et al. [296], the article selection process of the review is detailed in supplemental material. [file 7255497.f1.docx]

**Supplementary methods**

**Search strategy:**

Three medical research librarians developed literature search strategies using a list of keywords provided by the principle investigators. Searches were conducted in EMBASE, PubMed, and Web of Science (WOS). Using a similar modular search strategy to that described in Ranna, et. al, (2019) [1], the librarians created search statements for chemotherapy, mucositis, oxidative Stress, and anatomical terms. Furthermore, filter statements were created to remove articles about clinical studies, conditions not related to mucositis, and certain publication types. See Table S1-3 for a list of each module and the corresponding search string of EMBASE, PubMed, and WOS.

The medical librarians input the search results into an EndNote library and identified duplicate articles. Please see Table S4 for the final search statements used in EMBASE, PubMed, and WOS, respectively.

**Tables:**

**Table S1: Module and search string in EMBASE**

| Statement Module | Emtree | Entry Terms | Ssearch string |
| --- | --- | --- | --- |
| Chemotherapy | chemotherapy | Chemotherapeutics | 'chemotherapy'/exp OR chemotherapeutics:ti,ab,kw |
| Mucositis | enteritis | acute haemorrhagic necrotising enteritis  acute hemorrhagic necrotizing enteritis  granulomatous enteritis  inflammatory enteropathy  intestin* inflammation | 'enteritis'/exp OR 'acute haemorrhagic necrotising enteritis':ti,ab,kw OR 'acute hemorrhagic necrotizing enteritis':ti,ab,kw OR 'granulomatous enteritis':ti,ab,kw OR 'inflammatory enteropathy':ti,ab,kw OR 'intestin* inflammation':ti,ab,kw |
| Oxidative Stress | Oxidative Stress | oxidant stress  oxidative stress*  stress, oxidative | 'oxidative stress'/exp OR 'oxidant stress':ti,ab,kw OR 'oxidative stress*':ti,ab,kw OR 'stress, oxidative':ti,ab,kw |

**Table S2: Module and search string in PubMed**

| Statement Module | MeSH | Entry Terms | Ssearch string |
| --- | --- | --- | --- |
| Chemotherapy | Antineoplastic Agents | Antineoplastic*  Agent*, Antineoplastic  Antineoplastic Agent*  Anticancer Agent*  Agent*, Anticancer  Antineoplastic Drug*  Drug*, Antineoplastic  Antitumor Drug*  Drug*, Antitumor  Antitumor Agent*  Agent*, Antitumor  Cancer Chemotherapy Agent*  Agent*, Cancer Chemotherapy  Chemotherapy Agent*, Cancer  Cancer Chemotherapy Drug*  Drug*, Cancer Chemotherapy  Chemotherapeutic Anticancer Agent*  Agent*, Chemotherapeutic Anticancer  Chemotherapeutic Anticancer Drug*  Drug*, Chemotherapeutic Anticancer  Chemotherapy Drug*, Cancer | (((((((((((((((((((((Antineoplastic Agents[Title/Abstract]) OR (Antineoplastic*[Title/Abstract])) OR (Agent*, Antineoplastic[Title/Abstract])) OR (Antineoplastic Agent*[Title/Abstract])) OR (Anticancer Agent*[Title/Abstract])) OR (Agent*, Anticancer[Title/Abstract])) OR (Antineoplastic Drug*[Title/Abstract])) OR (Drug*, Antineoplastic[Title/Abstract])) OR (Antitumor Drug*[Title/Abstract])) OR (Drug*, Antitumor[Title/Abstract])) OR (Antitumor Agent*[Title/Abstract])) OR (Agent*, Antitumor[Title/Abstract])) OR (Cancer Chemotherapy Agent*[Title/Abstract])) OR (Agent*, Cancer Chemotherapy[Title/Abstract])) OR (Chemotherapy Agent*, Cancer[Title/Abstract])) OR (Cancer Chemotherapy Drug*[Title/Abstract])) OR (Drug*, Cancer Chemotherapy[Title/Abstract])) OR (Chemotherapeutic Anticancer Agent*[Title/Abstract])) OR (Agent*, Chemotherapeutic Anticancer[Title/Abstract])) OR (Chemotherapeutic Anticancer Drug*[Title/Abstract])) OR (Drug*, Chemotherapeutic Anticancer[Title/Abstract])) OR (Chemotherapy Drug*, Cancer[Title/Abstract]) |
| Mucositis | Mucositis | Mucositide* | ("Mucositis"[Mesh]) OR (Mucositide*[Title/Abstract]) |
| Anatomical terms | Intestines | Intestin* | ("Intestines"[Mesh]) OR (Intestin*[Title/Abstract]) |
| Oxidative Stress | Oxidative Stress | Oxidative Stresse*  Stress, Oxidative  Antioxidative Stress*  Stress, Antioxidative  Stress, Anti-oxidative  Oxidative Damage*  Damage*, Oxidative  Stress Injury, Oxidative  Oxidative Injur*  Injury, Oxidative  Oxidative Cleavage*  Cleavage, Oxidative  Oxidative DNA Damage*  DNA Damage, Oxidative  Damage, Oxidative DNA  DNA Oxidative Damage*  Damage, DNA Oxidative  Oxidative Damage, DNA  Oxidative and Nitrosative Stress  Oxidative Nitrative Stress*  Nitrative Stress, Oxidative  Stress, Oxidative Nitrative  Stress*, Nitro-Oxidative | ("Oxidative Stress"[Mesh]) OR ((((((((((((((((((((((Antioxidative Stress*[Title/Abstract]) OR (Stress, Antioxidative[Title/Abstract])) OR (Oxidative Stresse*[Title/Abstract])) OR (Stress, Anti-oxidative[Title/Abstract])) OR (Oxidative Damage*[Title/Abstract])) OR (Damage*, Oxidative[Title/Abstract])) OR (Stress Injury, Oxidative[Title/Abstract])) OR (Oxidative Injur*[Title/Abstract])) OR (Injury, Oxidative[Title/Abstract])) OR (Oxidative Cleavage*[Title/Abstract])) OR (Cleavage, Oxidative[Title/Abstract])) OR (Oxidative DNA Damage*[Title/Abstract])) OR (DNA Damage, Oxidative[Title/Abstract])) OR (Damage, Oxidative DNA[Title/Abstract])) OR (DNA Oxidative Damage*[Title/Abstract])) OR (Damage, DNA Oxidative[Title/Abstract])) OR (Oxidative Damage, DNA[Title/Abstract])) OR (Oxidative[Title/Abstract] AND Nitrosative Stress[Title/Abstract])) OR (Oxidative Nitrative Stress*[Title/Abstract])) OR (Nitrative Stress, Oxidative[Title/Abstract])) OR (Stress, Oxidative Nitrative[Title/Abstract])) OR (Stress*, Nitro-Oxidative[Title/Abstract])) |

**Table S3: Module and search string in Web of Science**

| Statement Module | Emtree | Entry Terms | Ssearch string |
| --- | --- | --- | --- |
| Chemotherapy | chemotherapy | Chemotherapeutic* | TS=(chemotherapy) OR TS=(Chemotherapeutic*) |
| Mucositis | Mucositis | Mucositide* | TS=(Mucositis) OR TS=(Mucositide*) |
| Oxidative Stress | Oxidative Stress | oxidant stress  oxidative stress*  stress, oxidative | TS=(Oxidative Stress) OR TS=(oxidant stress) OR TS=(oxidative stress*) OR TS=(stress, oxidative) |
| Anatomical terms | Intestines | Intestin* | TS=(Intestines) OR TS=(Intestin*) |

**Table S2: Complete search entries**

| Search | Query |
| --- | --- |
| Complete search statement in **EMBASE** | ('enteritis'/exp OR 'acute haemorrhagic necrotising enteritis':ti,ab,kw OR 'acute hemorrhagic necrotizing enteritis':ti,ab,kw OR 'granulomatous enteritis':ti,ab,kw OR 'inflammatory enteropathy':ti,ab,kw OR 'intestin* inflammation':ti,ab,kw) AND ('chemotherapy'/exp OR chemotherapeutics:ti,ab,kw) AND ('oxidative stress'/exp OR 'oxidant stress':ti,ab,kw OR 'oxidative stress*':ti,ab,kw OR 'stress, oxidative':ti,ab,kw) |
| Complete search statement in **PubMed** | (("Oxidative Stress"[Mesh]) OR ((((((((((((((((((((((Antioxidative Stress*[Title/Abstract]) OR (Stress, Antioxidative[Title/Abstract])) OR (Oxidative Stresse*[Title/Abstract])) OR (Stress, Anti-oxidative[Title/Abstract])) OR (Oxidative Damage*[Title/Abstract])) OR (Damage*, Oxidative[Title/Abstract])) OR (Stress Injury, Oxidative[Title/Abstract])) OR (Oxidative Injur*[Title/Abstract])) OR (Injury, Oxidative[Title/Abstract])) OR (Oxidative Cleavage*[Title/Abstract])) OR (Cleavage, Oxidative[Title/Abstract])) OR (Oxidative DNA Damage*[Title/Abstract])) OR (DNA Damage, Oxidative[Title/Abstract])) OR (Damage, Oxidative DNA[Title/Abstract])) OR (DNA Oxidative Damage*[Title/Abstract])) OR (Damage, DNA Oxidative[Title/Abstract])) OR (Oxidative Damage, DNA[Title/Abstract])) OR (Oxidative[Title/Abstract] AND Nitrosative Stress[Title/Abstract])) OR (Oxidative Nitrative Stress*[Title/Abstract])) OR (Nitrative Stress, Oxidative[Title/Abstract])) OR (Stress, Oxidative Nitrative[Title/Abstract])) OR (Stress*, Nitro-Oxidative[Title/Abstract]))) AND (((("Mucositis"[Mesh]) OR (Mucositide*[Title/Abstract])) AND (("Intestines"[Mesh]) OR (Intestin*[Title/Abstract]))) AND ((((((((((((((((((((((Antineoplastic Agents[Title/Abstract]) OR (Antineoplastic*[Title/Abstract])) OR (Agent*, Antineoplastic[Title/Abstract])) OR (Antineoplastic Agent*[Title/Abstract])) OR (Anticancer Agent*[Title/Abstract])) OR (Agent*, Anticancer[Title/Abstract])) OR (Antineoplastic Drug*[Title/Abstract])) OR (Drug*, Antineoplastic[Title/Abstract])) OR (Antitumor Drug*[Title/Abstract])) OR (Drug*, Antitumor[Title/Abstract])) OR (Antitumor Agent*[Title/Abstract])) OR (Agent*, Antitumor[Title/Abstract])) OR (Cancer Chemotherapy Agent*[Title/Abstract])) OR (Agent*, Cancer Chemotherapy[Title/Abstract])) OR (Chemotherapy Agent*, Cancer[Title/Abstract])) OR (Cancer Chemotherapy Drug*[Title/Abstract])) OR (Drug*, Cancer Chemotherapy[Title/Abstract])) OR (Chemotherapeutic Anticancer Agent*[Title/Abstract])) OR (Agent*, Chemotherapeutic Anticancer[Title/Abstract])) OR (Chemotherapeutic Anticancer Drug*[Title/Abstract])) OR (Drug*, Chemotherapeutic Anticancer[Title/Abstract])) OR (Chemotherapy Drug*, Cancer[Title/Abstract]))) |
| Complete search statement for Web of Science | (TS=(chemotherapy) OR TS=(Chemotherapeutic*) ) AND (TS=(Mucositis) OR TS=(Mucositide*)) AND (TS=(Oxidative Stress) OR TS=(oxidant stress) OR TS=(oxidative stress*) OR TS=(stress, oxidative)) AND (TS=(Intestines) OR TS=(Intestin*)) |

**Reference:**

[1] Ranna V, Cheng KKF, Castillo DA, et al. Development of the MASCC/ISOO clinical practice guidelines for mucositis: an overview of the methods. *Supportive Care in Cancer*. 2019;27(10):3933-3948. doi:10.1007/s00520-019-04891-1.
